# Multidrug-Resistant *Acinetobacter baumannii* Osteomyelitis from Iraq

**DOI:** 10.3201/eid1403.070128

**Published:** 2008-03

**Authors:** Jason J. Schafer, Julie E. Mangino

**Affiliations:** * Ohio State University Medical Center, Columbus, Ohio, USA; 1 Current affiliation: University of Pittsburgh Medical Center – St. Margaret Hospital, Pittsburgh, Pennsylvania, USA.

**Keywords:** *Acinetobacter baumannii*, tigecycline, osteomyelitis, multidrug resistance, letter

## Abstract

Multidrug-Resistant *Acinetobacter baumannii* Osteomyelitis from Iraq

**To the Editor:** *Acinetobacter baumannii* identified in military settings is commonly multidrug resistant (MDR) (*1*–*3*). Tigecycline displays *A. baumannii* activity, but clinical experience is limited. We report a case of probable osteomyelitis caused by MDR *A. baumannii* and treated with tigecycline.

A 55-year-old man was transporting soldiers in Iraq when he sustained a grenade injury, in which material entered his anterior thigh and created a large posterolateral hip exit wound and an open left subtrochanteric femur fracture. He was flown to Germany; his wound was debrided, and the fracture was stabilized with an external fixator along with pins to his ilium and proximal and distal femur. A wound vacuum covered the exposed bones within the large soft tissue defect. He was stable upon transfer to our hospital 14 days after the injury; leukocyte count was 16,000/μL (reference range 4.5–11,000/μL), and erythrocyte sedimentation rate (ESR) was 44 mm/h (reference range 0–19 mm/h); blood cultures were not obtained. Plain radiographs showed an open femur fracture with gas in the soft tissue, shrapnel, and a gross deformity of the left iliac wing. ^111^Indium-labeled leukocyte imaging confirmed increased activity in the left acetabulum, femoral neck, and surrounding soft tissue. Two days after his arrival, the external fixator (except for 1 pin in the distal shaft and 1 in the proximal femur) was removed, and an open reduction and internal fixation (ORIF) of the femur was performed. A cephalomedullary femoral rod and hip screw and 60 tobramycin-impregnated beads were placed into the hip joint; a wound vacuum was placed over the defect. A deep sample of the iliac wing was obtained, ground into a homogenate, placed aseptically on media, and observed for microbial growth; both coagulase-negative *Staphylococcus* and gram-negative rods grew in 1 culture. Both were considered pathogens of probable osteomyelitis based on exposed periosteum. Treatment with vancomycin plus ciprofloxacin was begun. After the gram-negative rods were identified as MDR *A. baumannii,* tigecycline (MIC 1.5) was substituted for ciprofloxacin (MIC>2). *A. baumannii* was susceptible to tobramycin (MIC<2), intermediate to imipenem (MIC 8), and resistant to all other agents tested (Microscan, Dade Behring Company, Deerfield, IL, USA). Tigecycline susceptibility was performed by Etest (AB Biodisk, Solna, Sweden); breakpoints were inferred from available literature for *Enterobacteriaceae* (<2.0 is susceptible) as no current Clinical Laboratory Standards Institute breakpoints are established (*4*). Susceptibility testing for *Staphylococcus* spp. was not performed; tigecycline’s role in treating the staphylococci in this setting was not determined because vancomycin was also used.

Postoperatively, leukocyte count returned to normal, wound drainage decreased, and a computed tomographic scan showed appropriate femur alignment with progressive heterotopic bone in the ilium. The patient was transferred to our rehabilitation facility and continued on vancomycin and tigecycline. Two weeks after the ORIF (hospital day 38), the wound vacuum was removed, a split-thickness skin graft was placed, and the patient was discharged. He returned to our infectious diseases clinic 2 weeks later; ESR was 12; tigecycline and vancomycin were stopped after 43 days. The probable osteomyelitis of the femur and ilium was resolved by standard clinical and radiologic parameters.

Tigecycline has displayed activity against many MDR pathogens, including *A. baumannii* in vitro (*4*), although recent investigations have demonstrated resistance and inconsistent susceptibility patterns (*5*). Clinical management of *A. baumannii* bone infections in humans has not been well established. In an experimental animal model of methicillin-resistant *S. aureus,* tigecycline showed adequate bone concentration with microbial clearance in 90% and 100% of patients who received tigecycline and tigecycline plus rifampin, respectively (*6*). This suggests that tigecycline may have also been useful for the coagulase-negative staphylococci identified in this patient and could have been considered as the sole treatment agent.

Tigecycline concentration in bone was also evaluated in an experimental rat model and a single-dose human study (*7*,*8*). The rat model showed an area under the curve in bone ≈250× higher than plasma (*7*). The investigation in humans could not duplicate these results; the discrepancy was attributed to either tight binding of tigecycline to bone or poor extraction methods (*8*). The testing method used in previous animal models was recently adapted for human use and has suggested increased sensitivity (*9*). An assessment of human bone concentrations after multiple tigecycline doses may be necessary to determine the potential role in osteomyelitis management.

Tobramycin bone and surrounding tissue concentrations have been demonstrated after tobramycin-impregnated beads were placed in animals and humans with open fractures or chronic osteomyelitis (*10*). The role of tobramycin beads is not established for osteomyelitis, but use is common. Their contribution to this patient's outcome is difficult to assess because *A. baumannii* was also susceptible to tobramycin.

Cases of *A. baumannii* osteomyelitis have been documented recently, but isolates were susceptible to other agents; none were treated with tigecycline (*3*). The role of tigecycline for osteomyelitis with MDR *A. baumannii* requires further study.

J.E.M. received research funding from Wyeth to perform synergy testing with tigecycline.
